# A 30 Gb/s PAM4 underwater wireless laser transmission system with optical beam reducer/expander

**DOI:** 10.1038/s41598-019-45125-y

**Published:** 2019-06-13

**Authors:** Wen-Shing Tsai, Hai-Han Lu, Hsiao-Wen Wu, Chung-Wei Su, Yong-Cheng Huang

**Affiliations:** 1 Department of Electrical Engineering, Ming Chi University of Technology, New Taipei City, 243 Taiwan; 2 Institute of Electro-Optical Engineering, National Taipei University of Technology, Taipei, 106 Taiwan; 3 Department of Electronic Engineering, Tung Nan University, New Taipei City, 222 Taiwan

**Keywords:** Fibre optics and optical communications, Diode lasers

## Abstract

We have, so far as we know, proposed and demonstrated the first 30 Gb/s four-level pulse amplitude modulation (PAM4) underwater wireless laser transmission (UWLT) system with an optical beam reducer/expander over 12.5-m piped underwater channel/2.5-m high-turbidity harbour underwater channel. In piped underwater links, the performances of PAM4 UWLT systems get better with beam reduction given a small amount of light absorbed by the piped water. In highly turbid harbour underwater links, the performances of PAM4 UWLT systems get better with beam expansion given a large amount of scattered light received by the optical receiver. The effect of high-turbidity harbour water that induces scattering angle (beam divergence) on beam diameter is analyzed and optimised to enhance the transmission performances. This proposed PAM4 UWLT system, which uses an optical beam reducer/expander, provides a practical choice for high transmission capacity and considerably develops clarity and high-turbidity scenarios. It presents promising features for affording a high-transmission-rate underwater optical wireless transmission and opening an access to accelerate wide applications of UWLT systems.

## Introduction

Underwater wireless laser transmission (UWLT) is a promising contributor to high-capacity underwater transmissions. UWLT systems are gaining considerable attention because of their numerous applications^1–8^, such as ecological supervising, coastwise investigation, and underwater petroleum exploration. However, the performances of UWLT systems are limited by the fact that light propagation in water is attenuated by absorption and scattering^9^, which are affected by power transfer during interaction with water and particulate matter density. The effects of absorption in piped water and scattering in high-turbidity harbour water worsen the performances of UWLT systems. A high-transmission-rate UWLT system with an acceptable underwater link is required to meet the demands of various requests. In 2008, William *et al*. used the return-to-zero (RZ) on-off-keying technology to modulate a 405-nm laser diode (LD), which demonstrates an UWLT system with a bit rate of 500 Kb/s over a distance of 3.66 m^10^. Oubei *et al*. changed the data format to the orthogonal frequency-division multiplexing (OFDM) to enhance the data rate to 4.8 Gb/s with lengthened distance over 5.4 m^11^. Xu *et al*. further used the 32-quadrature amplitude modulation (QAM)-OFDM to increase UWLT capacity up to 4.88 Gb/s within a distance of 6 m^12^. Chen *et al*. changed the blue LD to a green LD at 520 nm to demonstrate the UWLT link at 5.5 Gb/s with a bit error rate (BER) of 2.9 × 10^−3^ ^13^. UWLT in excess of 10 Gb/s was not realized until 2018, when Huang *et al*. used the filtered multi-carrier OFDM encoding format. This technology suppresses the inter-carrier interference induced crosstalk between OFDM subbands to increase the UWLT data rate up to 14.8 Gb/s^14^.

An optical beam reducer/expander that can reduce/expand the beam size is used in free-space optical (FSO) transmissions to enhance the overall qualities^15,16^. UWLT systems are similar to FSO systems because both utilize laser beams to transport optical signals between the transmitting and receiving sides. Thus, optical beam reducer/expander is anticipated to better the overall UWLT system performances in various water types, such as piped water and high-turbidity harbour water^17–19^. In piped underwater links, absorption is the primary contributor, and a low scattering coefficient frees the laser beam from divergence. With an optical beam reducer, the performances of UWLT systems in piped water can be gotten better given a small amount of light absorbed by the piped water. In high-turbidity harbour underwater links, the density of dissolved particles is high, which affects scattering. With an optical beam expander, the performances of UWLT systems in high-turbidity harbour water can be gotten better as a result of a large amount of scattered light received by the optical receiver. Therefore, an optical beam reducer/expander is a favourable contributor for enhancing the UWLT systems’ transmission performances. This illustration presents a 30 Gb/s four-level pulse amplitude modulation (PAM4) UWLT system with an optical beam reducer/expander over 12.5-m piped underwater channel/2.5-m high-turbidity harbour underwater channel. To the best of our knowledge, our demonstration is the leading to utilize a 488-nm blue-light LD transmitter in a 30 Gb/s PAM4 UWLT system through piped water or highly turbid harbour water. The results show that in piped/highly turbid harbour underwater links, the BER and eye diagrams performances get better with beam reduction/expansion. A previous study demonstrated the practicability of setting up an 8 m/9.6 Gbps 16-QAM-OFDM UWLT system on the basis of a 405-nm blue-light LD transmitter with a two-stage injection locking technique^20^. However, this application requires a costly arbitrary waveform generator and a complex two-stage injection-locked technique with elaborate wavelength detuning. The feasibility of establishing an underwater fibre-wireless communication over 50-m step-index plastic optical fibre (SI-POF) transport with 2-m underwater wireless channel has also been proven^21^. Nevertheless, the transmission rate of underwater fibre-wireless communications (1.71 Gb/s) is limited by the bandwidth of SI-POF. Another study has demonstrated the possibility of constructing a 16-Gb/s/10-m PAM4 UWLT system on the basis of a 488-nm blue-light LD transmitter with light injection and optoelectronic feedback techniques^22^. Nonetheless, the performances of PAM4 UWLT systems can be further improved using an optical beam reducer. This demonstration presents a 30 Gb/s PAM4 UWLT system with optical beam reducer/expander that outperforms existing UWLT systems due to its ability to provide a high-transmission-rate underwater channel.

## Results

### The optical power-driving current (P-I) curve and the optical spectra of the blue-light LD

**Figure 1:**
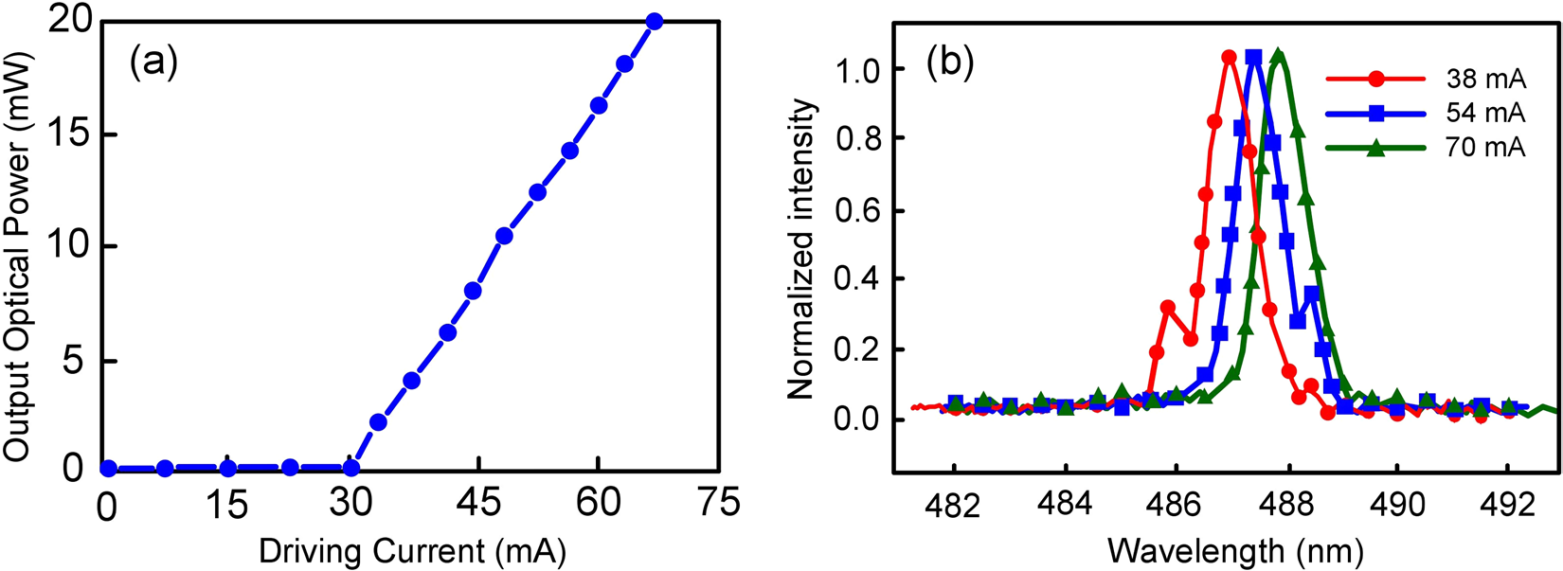
(**a**) P-I curve of the blue-light LD with a threshold current of 30 mA and a slope efficiency of approximately 50 mW/mA. (**b**) Optical spectra of the blue-light LD at room temperature (25 °C) under various driving currents.

Figure 1(a) shows the optical power-driving current (P-I) curve of the blue-light LD with a threshold current of 30 mA and a slope efficiency of approximately 50 mW/mA. A maximum optical output power of 20 mW is attained at a driving current of 70 mA. Figure 1(b) presents the optical spectra of the blue-light LD at room temperature (25 °C) under various driving currents. The peak emission wavelengths at driving currents of 38, 54 and 70 mA are around 487.15, 487.54 and 488 nm, respectively. The peak emission wavelength shifts to a longer wavelength to a small extent with an increase in driving current.

### The frequency responses of the LD-based PAM4 UWLT systems and the gain curve of the linear equalizer

**Figure 2:**
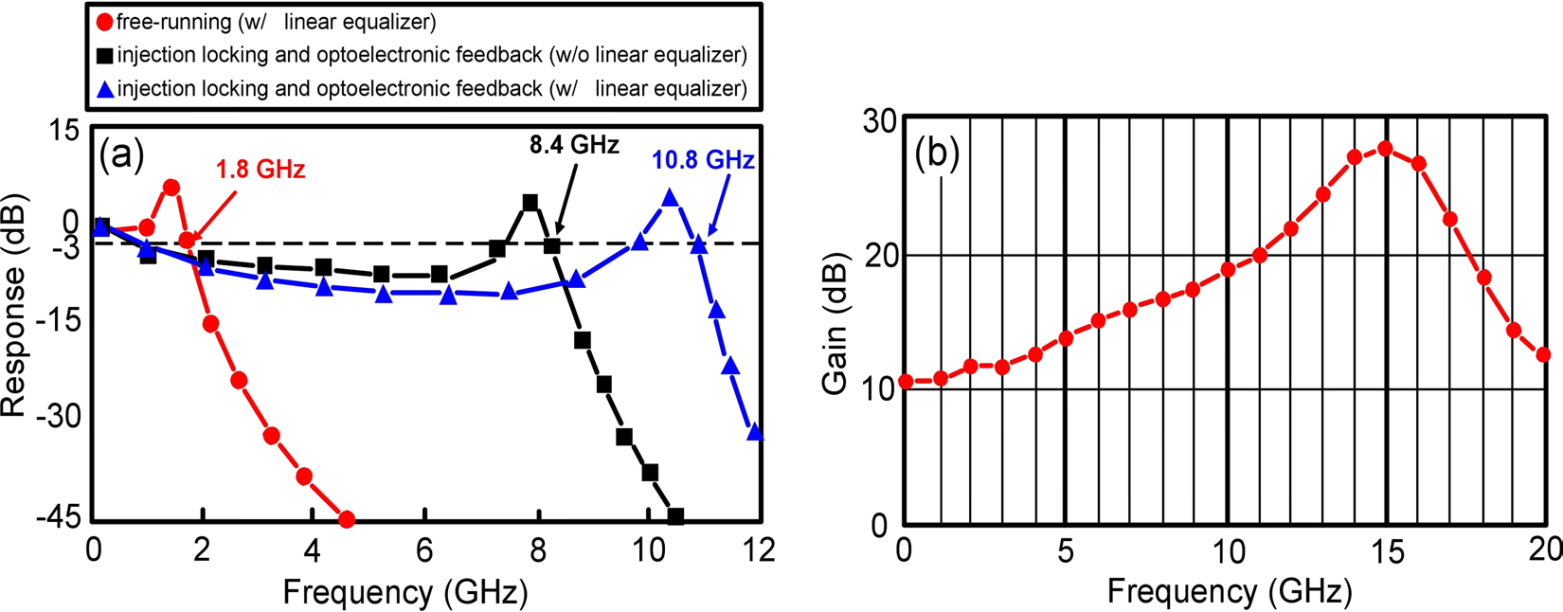
(**a**) Frequency response of the LD-based PAM4 UWLT systems for different states of free-running (with a linear equalizer), injection locking and optoelectronic feedback (without a linear equalizer), and injection locking and optoelectronic feedback (with a linear equalizer). (**b**) Gain curve of the linear equalizer.

Figure 2(a) shows the frequency responses of the LD-based PAM4 UWLT systems for different states of free-running (with a linear equalizer at receiving end), injection locking and optoelectronic feedback (without a linear equalizer at receiving end), and injection locking and optoelectronic feedback (with a linear equalizer at receiving end). For free-running (with a linear equalizer), a 1.8-GHz 3-dB bandwidth is acquired. For LD with injection locking and optoelectronic feedback (with a linear equalizer), a 10.8-GHz 3-dB bandwidth is acquired. The frequency response is characterised by a drop in the middle frequencies and a resonance peak in high frequencies to attain a considerable 3-dB bandwidth enhancement. This 3-dB bandwidth enhancement (from 1.8 GHz to 10.8 GHz) results from the effects of injection locking and optoelectronic feedback. Injection locking and optoelectronic feedback techniques considerably increase the photon density, which leads to a remarkable bandwidth enhancement^23,24^. Injection locking and optoelectronic feedback are the most promising techniques to conquer the bandwidth limitation. This result reveals that, with the adoption of linear equalizer at receiving end, a 488-nm blue-light LD transmitter with injection locking and optoelectronic feedback techniques is satisfactory for a 30 Gb/s PAM4 UWLT system [10.8 × $\sqrt{2}$ (as transmission rate is $\sqrt{2}$ times bandwidth) × 2 (PAM4 modulation) >30].

Figure 2(b) shows the gain curve of the linear equalizer. Linear equalization signifies an operation designed to improve the levels of high frequencies (10–17.6 GHz) compared with the levels of low frequencies (DC–10 GHz). A linear equalizer compensates for the frequency response (especially for high frequencies) and enhances the transmission rate of PAM4 UWLT systems^25,26^.

### BER performance of 30 Gb/s PAM4 UWLT systems with/without an optical beam reducer/expander over 12.5-m piped underwater channel/2.5-m high-turbidity harbour underwater channel

**Figure 3:**
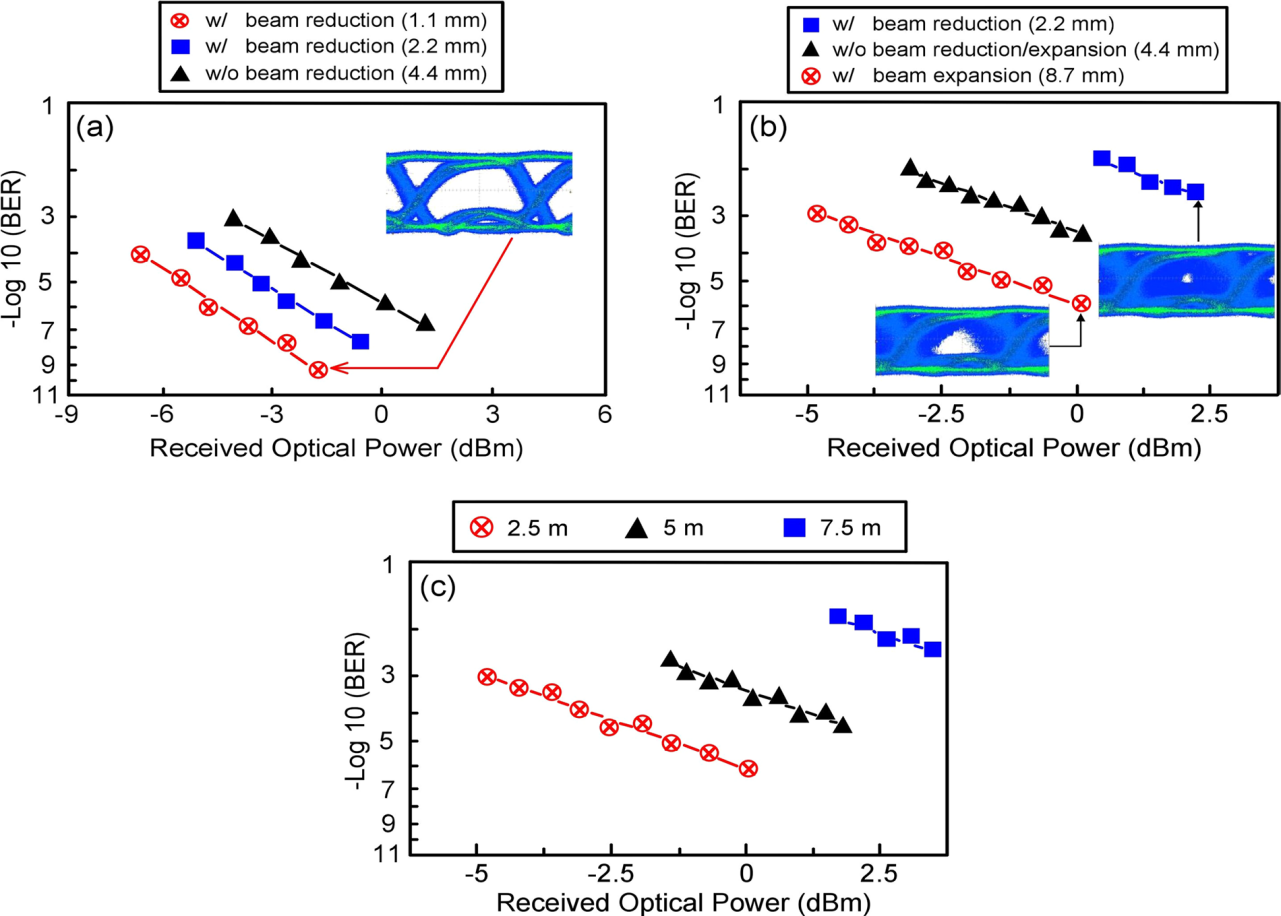
BER performance of 30 Gb/s PAM4 UWLT systems (**a**) with/without an optical beam reducer over 12.5-m piped underwater channel, (**b**) with/without an optical beam reducer/expander over 2.5-m high-turbidity harbour underwater channel, and (**c**) with an optical beam expander that expands the beam diameter to 8.7 mm over 2.5-m, 5-m, and 7.5-m high-turbidity harbour underwater channel.

Figure 3(a) presents the BER performance of the 30 Gb/s PAM4 UWLT systems with/without an optical beam reducer over 12.5-m (2.5 m × 5) piped underwater channel. With piped water, BER performance increases as beam size decreases. The BER is 2.4 × 10^−7^ without an optical beam reducer (with 4.4 mm beam diameter), but it improves to 10^−9^ with an optical beam reducer that reduces the beam diameter to 1.1 mm. Such BER performance enhancement is primarily attributed to the effect of beam reduction. Moreover, with 4.4 mm beam diameter, the received optical power is approximately 1.4 dBm to compensate for the decline in optical signal-to-noise ratio (OSNR). Nevertheless, this compensation is restricted and only 2.4 × 10^−7^ BER operation is attained. Furthermore, for the state of identical received optical power, the optical power of LD transmitter with 4.4 mm beam diameter is higher than one with 1.1 mm beam diameter, which leads to poor OSNR and worse BER performance. Figure 3(a) also shows the eye diagram of 15 Gb/s non-RZ (NRZ) signal with an optical beam reducer that further reduces the beam diameter. Open eye diagram is acquired as the beam diameter further reduces to 1.1 mm.

Overall underwater losses can be expressed by the attenuation coefficient *c*(λ) (=*a*(λ) + *s*(λ)) at a certain wavelength λ. *a*(λ) and *s*(λ) are the absorption and the scattering coefficients, respectively, changing with different water types and optical wavelengths. In piped water, the coefficients of *a*(λ), *s*(λ) and *c*(λ) are 0.053 (m^−1^), 0.006 (m^−1^) and 0.059 (m^−1^) (at 488 nm), respectively^27–29^. Given that *a*(λ) offers approximately 9 times the value of *s*(λ) (0.053/0.006 ~ 9), absorption is a significant contributor in piped underwater links. With an optical beam reducer that reduces the beam diameter to 1.1 mm, the performances of the PAM4 UWLT systems improve substantially given a small amount of light absorbed by the piped water (a large amount of light accumulated by the fibre collimator and received by the optical receiver). A smaller beam diameter causes lower absorption and higher OSNR, brings on higher amount of light received by the optical receiver, and thereby results in lower BER. However, the optical beam reducer expands the beam divergence because of the product conservation of beam diameter and beam divergence^30^. For a piped underwater link, given that the ratio of the scattered light is small, a smaller beam diameter with a larger beam divergence brings on a smaller amount of light scattered by the piped water. Thus, the advantages of small beam width are attained.

Figure 3(b) shows the BER performance of 30 Gb/s PAM4 UWLT systems with/without an optical beam reducer/expander over 2.5-m high-turbidity harbour underwater channel. In highly turbid harbour underwater links, BER performance increases as beam size increases. With an optical beam reducer that reduces the beam diameter to 2.2 mm, BER declines to 6.5 × 10^−3^. Without an optical beam reducer/expander (with 4.4 mm beam diameter), BER reaches 7.4 × 10^−4^. With an optical beam expander that expands the beam diameter to 8.7 mm, BER improves to 10^−6^. The received optical power is around 2.3 dBm to compensate for the decline in OSNR with a 2.2-mm beam diameter. Nonetheless, the compensation is restricted, and a BER of only 6.5 × 10^−3^ is attained. Moreover, for the state of identical received optical power, the optical power of the LD transmitter with 2.2 mm beam diameter is higher than one with 8.7 mm beam diameter, which leads to poor OSNR and worse BER performance.

Figure 3(b) also shows the eye diagrams of 15 Gb/s NRZ signal with an optical beam reducer/expander. A close eye diagram is observed for beam reduction (2.2 mm). However, a somewhat clear eye diagram is observed for beam expansion (8.7 mm). In highly turbid harbour water, the coefficients of *a*(λ), *s*(λ) and *c*(λ) are 0.427 (m^−1^), 2.098 (m^−1^) and 2.525 (m^−1^) (at 488 nm), respectively^27–29^. Given that *s*(λ) offers 4.91 times the value of *a*(λ) (2.098/0.427 = 4.91), scattering is the primary contributor in highly turbidity harbour underwater links. With an optical beam expander that expands the beam diameter to 8.7 mm, the performances of the PAM4 UWLT systems are enhanced as a result of higher amount of scattered light received by the optical receiver. Given that beam divergence is inversely proportional to the beam diameter, a reduced beam divergence occurs with an optical beam expander^30^. A larger beam diameter that follows a smaller beam divergence contributes more scattered light accumulated by the fibre collimator and received by the optical receiver, and leads to higher OSNR and lower BER. However, a larger beam diameter accompanies a larger absorption. For a highly turbid harbour underwater link, given that the ratio of absorbed light is small, a smaller beam divergence with a larger beam diameter brings on a smaller amount of light absorbed by the highly turbid harbour water. Thus, the advantages of large beam width are attained.

The BER performance of 30 Gb/s PAM4 UWLT systems with an optical beam expander that expands the beam diameter to 8.7 mm over 2.5-m, 5-m, and 7.5-m high-turbidity harbour underwater channel is presented in Fig. 3(c). BER increases with the increase in underwater link. BER reaches 10^−6^ over 2.5-m underwater channel and 6.4 × 10^−5^ over 5-m underwater channel. It degrades to 4.2 × 10^−3^ over 7.5-m underwater channel. Such BER performance degradation arises from the decrease in OSNR because of underwater link over 7.5-m underwater channel and the laser beam misalignment between the transmitting and receiving sides. A longer high-turbidity harbour underwater transmission results in higher attenuation (higher scattering), which leads to poor OSNR and worse BER. Moreover, laser beam alignment between transmitting and receiving sides is critical to the performances of high-transmission-rate PAM4 UWLT systems^31–33^. BER increases with the increase in laser beam misalignment. Excellent directing and alignment techniques are needed to attain good transmission performances. System designers should address the maximum/tolerable laser beam misalignment to achieve good transmission quality.

In this work, which employed an innovative high-transmission-rate PAM4 UWLT system design, the beam diameter of an injected blue-light LD transmitter detuned by beam reduction/expansion is employed to carry the PAM4 data stream through piped/highly turbid harbour underwater links. The performances of 30 Gb/s PAM4 UWLT systems with reduced/expanded beam diameter are discussed. The effect of high-turbidity harbour water that induced beam divergence (scattering angle) is analyzed and optimized to enhance the transmission performances. A 30 Gb/s PAM4 UWLT system based on a 488-nm blue-light LD transmitter with an optical beam reducer/expander over 12.5-m piped/2.5-m high-turbidity harbour underwater channel is successfully demonstrated. In piped underwater links, the overall performances of the PAM4 UWLT systems get better with beam reduction as a result of a small amount of light absorbed by the piped water. In highly turbid harbour underwater links, the performances of the proposed PAM4 UWLT systems get better with beam expansion as a result of a large amount of scattered light received by the optical receiver. This proposed PAM4 UWLT system affords the benefit of high transmission capacity, which is a promising characteristic that can accelerate the development of high-transmission-rate PAM4 UWLT systems.

## Discussion

**Figure 4:**
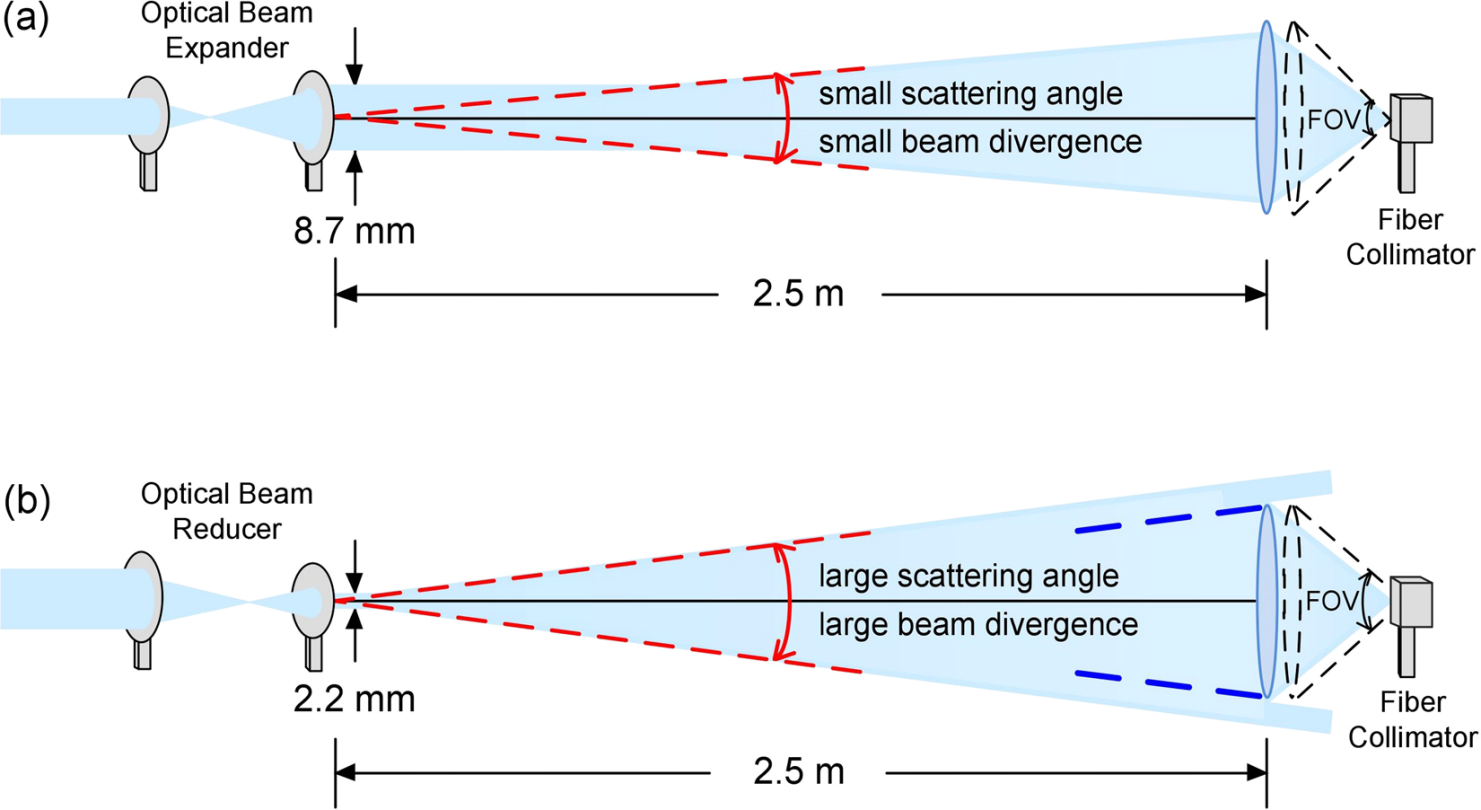
(**a**) For the state of small scattering angle (small beam divergence), the fibre collimator accumulates more scattered light. (**b**) For the state of large scattering angle (large beam divergence), the fibre collimator accumulates less scattered light.

In a highly turbid harbour underwater channel, it is vital to match the fibre collimator’s field of view of in receiver optics to maintain high transmission rates in PAM4 UWLT systems^34^. The key issue is to match the beam size to the fibre collimator’s receiving area to avoid large coupling loss and keep the high-turbidity harbour underwater link working. Since that the laser beam is very narrow and the fibre collimator’s receiving area is quite small, maintaining an underwater link for a qualified optical wireless connection is essential. Given a small scattering angle (small beam divergence), as illustrated in Fig. 4(a), then the fibre collimator accumulates more scattered light. Received optical power thereby increases, leading to better BER performance. Conversely, given a large scattering angle (large beam divergence), as illustrated in Fig. 4(b), the fibre collimator accumulates less scattered light, leading to an apparent decrease in received optical power and worse BER performance.

In highly turbid harbour underwater links, the attenuation coefficient *c*(λ) at 680 nm is lower than that at 488 nm^35^. With a low attenuation coefficient, the transmission qualities of PAM4 UWLT systems can be further enhanced as a result of a further small amount of light attenuated by the highly turbid harbour water. Therefore, the performances of PAM4 UWLT systems with optical beam expander are anticipated to be further improved by adopting a 680-nm red-light vertical-cavity surface-emitting laser (VCSEL) transmitter in highly turbid harbour underwater links. For the future extension of PAM4 UWLT systems, a 680-nm red-light VCSEL transmitter in a highly turbid harbour underwater link can be deployed to establish a high-transmission-rate PAM4 UWLT system, rather than a 488-nm blue-light LD transmitter.

## Methods

### Framework of the demonstrated 30 Gb/s PAM4 UWLT systems with an optical beam reducer/expander

**Figure 5:**
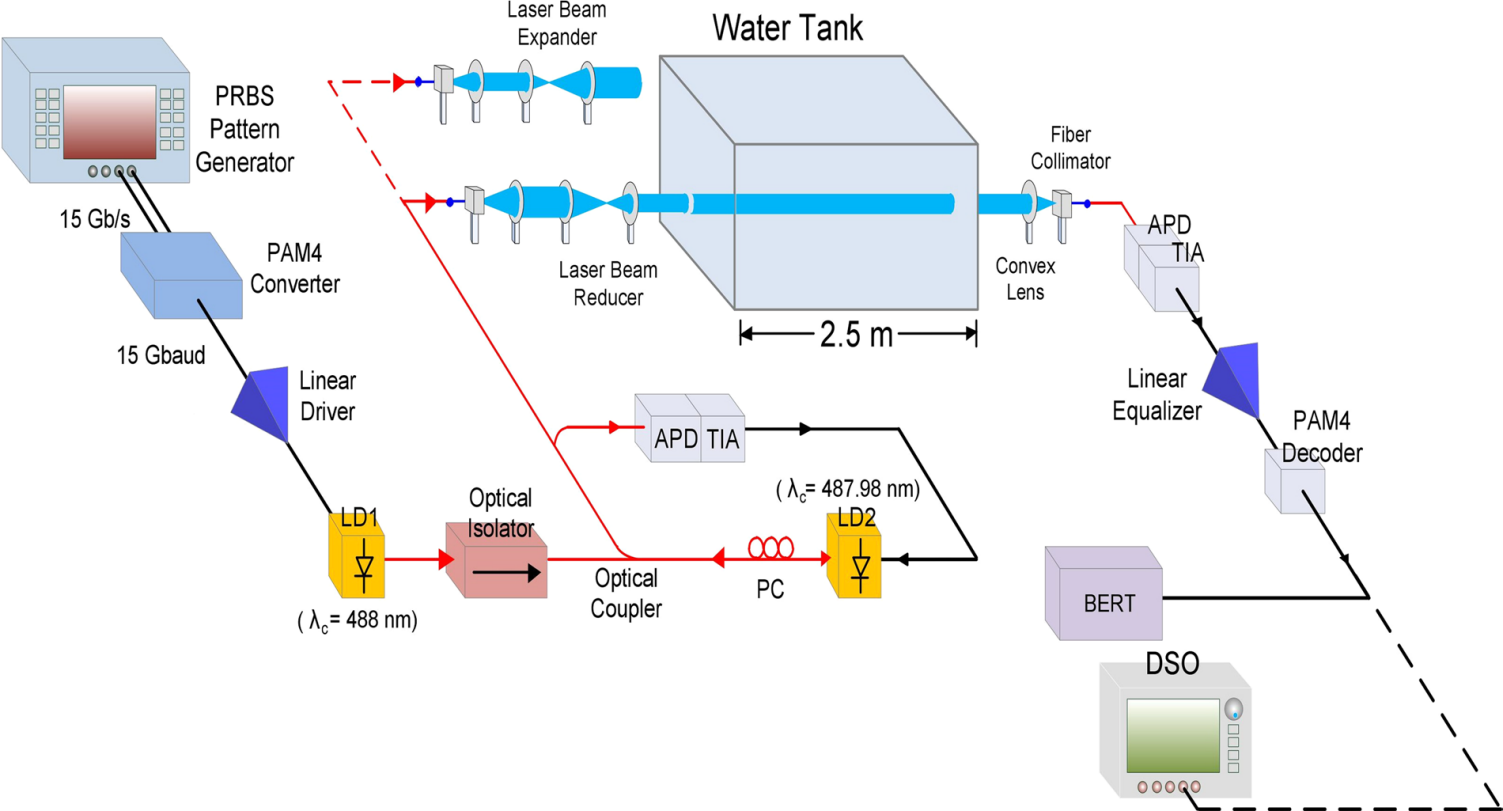
Framework of the demonstrated 30 Gb/s PAM4 UWLT systems with an optical beam reducer/expander.

Figure 5 illustrates the framework of the demonstrated 30 Gb/s PAM4 UWLT systems with an optical beam reducer/expander. A pseudorandom bit sequence (PRBS) pattern generator with two output channels generates two binary PRBS data streams with an aligned clock at 15 Gb/s with a length of 2^15^–1. The two binary PRBS data streams have amplitudes of 1.2 and 0.6 V, respectively. A PAM4 converter is used at the transmitting side to transform two 15 Gb/s NRZ signals into a 30 Gb/s (15 Gbaud) four-level PAM4 signal. The 30 Gb/s PAM4 signal is supplied to the LD1 after boosting by a linear driver. Given that PAM4 linearity is an important parameter, a linear driver with high-linearity is adopted to drive the PAM4 signal utilizing the linear region of the P-I curve of the LD1. If LD2 (slave laser) is modulated with a 30 Gb/s PAM4 signal, then PAM4 signal modulation will be high^36^. Nevertheless, low PAM4 signal modulation can be compensated by the boost of linear driver. Thus, the performances of the PAM4 UWLT systems influenced by low PAM4 signal modulation is restricted. A division of laser light is utilized for the optoelectronic feedback loop, and another division of laser light is utilized for the UWLT systems. The avalanche photodiode (APD) and linear trans-impedance amplifier (TIA) convert the laser light into a 30 Gb/s PAM4 signal to directly modulate the LD2. The laser light emanated from a fibre collimator is inputted into a convex lens with 50-mm focal length (for beam reduction)/25.4-mm focal length (for beam expansion). The resulting beam is supplied to an optical beam reducer/expander, transmitted through piped/highly turbid harbour underwater channel, supplied to a convex lens with 25.4-mm focal length (for beam reduction)/50-mm focal length (for beam expansion), and eventually guided into a fibre collimator with 350–700 nm operational wavelength range.

A 2.5 m-long water tank is loaded with piped water with a particle concentration < 0.1 g/m^3^ or high-turbidity harbour water with a particle concentration of 33.46 g/m^3^. Water turbidity is made with suspensions of Mg(OH)_2_ and Al(OH)_3_, by adding a commercial antacid preparation (Maalox)^37^. In piped underwater links, the underwater transmission distance is enhanced to 12.5 m (2.5 m × 5) with the adoption of plane mirrors on both sides of the water tank. In highly turbid harbour underwater links, however, the underwater transmission distance is only 2.5 m (without the adoption of plane mirrors on both sides of the water tank). Over 12.5-m piped/2.5-m high-turbidity harbour underwater channel, the laser light is detected and enhanced by an APD with TIA receiver. After electronic equalization by a linear equalizer, a 30 Gb/s PAM4 signal is converted into two 15 Gb/s NRZ signals using a PAM4 decoder. Thereafter, we use a BER tester to calculate the BER values and a digital storage oscilloscope to capture the 15 Gb/s NRZ eye diagrams. BER and NRZ eye diagrams are taken as performance metrics.

### Optical beam reducer/expander for beam reduction/expansion

**Figure 6:**
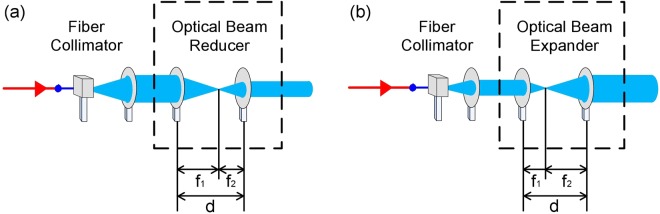
Optical beam reducer/expander that can reduce/expand the beam diameter.

Figure 6 shows the optical beam reducer/expander that can reduce/expand the beam diameter. An optical beam reducer [Fig. 6(a)]/expander [Fig. 6(b)] contains two convex lenses with unequal focal lengths denoted by *f*_1_ and *f*_2_. For optical beam reducer, *f*_1_ is longer than *f*_2_. As for optical beam expander, *f*_1_ is shorter than *f*_2_. For an optical beam reducer/expander, the split distance of two convex lenses (*d*) is identical to the summation of the focal lengths (*f*_*1*_ + *f*_2_). An optical beam reducer reduces a large beam diameter to a small one. By contrast, an optical beam expander expands a small beam diameter to a large one.
